# Strengths and Obstacles of Health Informatics and Health Information Management Education and Professions in Hail City, Kingdom of Saudi Arabia: A Qualitative Study

**DOI:** 10.7759/cureus.52619

**Published:** 2024-01-20

**Authors:** Anas Alhur, Bakheet Aldosari

**Affiliations:** 1 Health Informatics, College of Public Health and Health Informatics, University of Hail, Hail, SAU; 2 Health Informatics, King Abdulaziz Medical City, Riyadh, SAU

**Keywords:** obstacles, strengths, swot, digital health, health information management, health informatics

## Abstract

Health and higher education ministries in Saudi Arabia recognize the need for a highly qualified workforce specializing in health informatics and information management (HIIM). Therefore, KSA colleges and universities offer HIIM programs, health information systems, and health information technology. It is critical to investigate the HIIM professions and education in Hail City due to differences in these educational programs. This study examined HIIM professions and education in Hail City, Saudi Arabia, and provided strategies and recommendations. Based on a qualitative research approach, we interviewed 39 academicians, health informaticians, and other stakeholders in Hail City. The strengths, weaknesses, opportunities, threats (SWOT) framework was used to explore HIIM status and make recommendations. According to participants, HIIM Saudi professionals in Hail City have been growing and motivated, as have the university's undergraduate and postgraduate programs. Informants indicated several weaknesses, but many opportunities were found, including Saudi Vision 2030 and a new HIIM faculty at the University of Hail. According to our findings, relevant specialties control HIIM leadership while unspecialized academicians provide instruction. The extraordinary transmission of digital health in Saudi Arabia promises to enhance HIIM professions and education strengths and opportunities and reduce their weaknesses and threats.

## Introduction

The imperative for continuous education evaluation and improvement becomes increasingly pronounced in a world characterized by rapid advancements in knowledge and technology. This urgency is particularly salient in nations like Saudi Arabia, where pursuing higher education is paramount for societal progress. Saudi Arabia has made significant strides in fortifying its higher education system, aiming to equip individuals with high-quality education. Initiatives like those by King Saud University have played a pivotal role in this advancement [1,2], leading to a proliferation of educational institutions across the country. These institutions offer diverse degree programs in various disciplines, reflecting the evolving educational landscape.

Health informatics (HI) is a dynamic and interdisciplinary field that lies at the intersection of healthcare, information technology, and management. It can be broadly defined as the study and application of methods to collect, analyze, and use biomedical data, information, and knowledge for scientific research, problem-solving, and decision-making, all aimed at improving patient outcomes and healthcare efficiency [3]. This field is characterized by its interprofessional nature, involving a diverse range of experts from medical, IT, and administrative backgrounds working collaboratively to enhance healthcare delivery.

The American Medical Informatics Association (AMIA) provides a comprehensive description of health informatics and information management (HIIM). According to AMIA, HIIM encompasses a wide range of cognitive, information processing, and communication tasks that are essential in healthcare practices, education, and research, supported by the principles and advancements in information science and technology [4]. This perspective emphasizes the role of HIIM in streamlining healthcare operations, improving patient care, and fostering educational and research initiatives in health. Furthermore, the National Library of Medicine (NLM) accentuates the role of HI in the design, development, adoption, and application of IT-based innovations in the healthcare sector [5].

Acknowledging the importance of a qualified workforce in HIIM, both health and higher education ministries in Saudi Arabia are focusing on advancing healthcare practices and education through these disciplines [6,7]. Consequently, several universities nationwide have introduced HIIM programs, offering degrees in health informatics, health information systems management, and health information technology (HIT). Notably, only two master's programs in HIIM are currently available: the Master of Science in Health Informatics at King Saud bin Abdulaziz University for Health Sciences and the Executive Master of Health Informatics (E-Health) program at the University of Hail [8,9]. These programs cater to students interested in merging health sciences with computer science and emphasize the importance of collaboration among HI specialists and healthcare professionals from different areas [10,11].

Saudi Arabia has dramatically improved healthcare services by implementing e-health systems. As the coronavirus disease 2019 (COVID-19) pandemic intensified, skilled professionals have become even more necessary for efficiently managing and operating electronic health systems [12]. Therefore, Saudi Arabia accelerated digital health initiatives, with public and private sectors adopting various digital tools, platforms, and applications for COVID-19 screening, disease surveillance, contact tracing campaigns, and awareness campaigns - improving overall healthcare outcomes in Saudi Arabia [13].

HIMM faces global challenges despite its vital role in healthcare. Educators are tasked with the complex responsibility of developing curriculum content that not only aligns with various educational agendas and areas of expertise but also adapts to diverse teaching styles [14]. This challenge highlights the need for a detailed and thorough understanding of HI and its various aspects.

In the context of our study, this understanding is pivotal. It lays the groundwork for an in-depth exploration of the strengths and obstacles faced by HIIM education and professions, with a particular focus on Hail City, KSA. This unique setting offers a distinctive perspective on the dynamics of HIIM, enriching the broader discourse in this field. Our aim is to delve into these diverse perspectives of HI, examining how they interact and influence the development of HIIM. Through this exploration, we seek to contribute significantly to the ongoing discourse on optimizing HIIM to meet the evolving needs of the healthcare industry, particularly in settings that mirror the characteristics and challenges of Hail City.

## Materials and methods

Participants

This study involved professionals and students in the field of HIIM. The participant group comprised health informatics professionals, senior/mid-level management staff, and undergraduate and graduate students.

Phase 1 - identification and recruitment of key informants

In the initial phase of our study, we focused on identifying and recruiting key informants who possessed significant expertise in the field of HIIM. To ensure a comprehensive understanding of the HIIM landscape in Hail City, we established specific criteria for selecting these individuals. These criteria were as follows.

Professional Background and Experience

We targeted professionals who had a minimum of two years of experience in the HIIM sector. This included health informatics professionals, senior and mid-level management staff, and healthcare practitioners actively involved in informatics and information management.

Current Role and Responsibilities

Preference was given to individuals currently holding positions that directly interact with or influence the HIIM field. This encompassed roles such as IT project managers, department heads, healthcare IT senior managers, and academic professionals with a focus on HIIM.

Affiliation With Key Institutions

We specifically sought out individuals affiliated with major hospitals in Hail City and faculties or departments within the Hail University campus who are engaged in HIIM education or practice.

Contributions to the Field

Candidates who had contributed to the field through research, publications, or significant projects in HIIM were considered favorably. This helped in ensuring that our informants had a deep understanding and were influential in the field.

Diversity of Perspectives

To ensure a well-rounded view, we aimed for a diverse group of informants in terms of gender, educational background, and professional experience.

Using these criteria, we identified and approached potential key informants. The recruitment process involved reaching out to these individuals via email or phone, briefly outlining the purpose of our study and the nature of their expected contribution. We ensured that our selection encompassed a broad spectrum of professionals who could provide varied and in-depth insights into the HIIM education and profession in Hail City.

Phase 2 - key informant interviews

In the second phase of our study, we conducted in-depth interviews with the selected key informants to explore the challenges and experiences associated with HIIM in Hail City. The interviews were meticulously designed to elicit comprehensive and meaningful insights:

Development of the Interview Schedule

The interview schedule was developed based on a thorough review of existing literature and current issues in the HIIM field. It aimed to cover a range of topics relevant to our research objectives, including education, professional challenges, technological advancements, and policy impacts in HIIM.

Nature of Questions

The interview schedule included a balanced mix of open-ended and closed-ended questions. Open-ended questions were designed to encourage informants to share their experiences, perceptions, and insights in depth. Closed-ended questions were used to gather specific information and statistical data relevant to our study.

Pilot Testing

Prior to the actual interviews, the schedule was pilot-tested with a small group of professionals (not included in the main study) to ensure clarity and relevance of the questions. Feedback from this pilot test was used to refine the questions further.

Conducting the Interviews

Each interview lasted approximately 15 minutes and was conducted in a semi-structured format to allow for flexibility in responses while maintaining focus on the key topics. The interviews were carried out either in person or via teleconferencing, depending on the availability and preference of the informants.

Recording and Transcription

With the consent of the participants, all interviews were recorded to ensure accurate capture of the data. The recordings were then transcribed verbatim for subsequent analysis. This approach ensured that we could accurately capture and analyze the nuanced responses of the informants.

Ensuring Confidentiality

To maintain the confidentiality of the informants, all identifying information was removed from the transcripts, and data was handled in accordance with ethical research standards.

Phase 3 - data analysis

In the third phase of our study, we meticulously analyzed the collected data using NVivo software, a sophisticated tool designed by QSR International Pty Ltd. This software is renowned for its ability to facilitate in-depth qualitative data analysis, allowing researchers to identify and explore complex patterns within large datasets.

The data analysis process commenced with an initial exploratory phase. In this stage, we performed a preliminary review of the interview transcripts to familiarize ourselves with the data. This exploration was crucial for developing an understanding of the overall content and context of the responses.

Subsequently, we moved to the crystallization phase, a methodical process aimed at identifying recurring themes and patterns. This phase involved a detailed and thorough examination of the data, where we sought to uncover underlying themes that were repeatedly evident across different interviews.

To capture the richness and complexity of the data, we employed nonlinear coding techniques. This approach allowed us to recognize both anticipated themes (those we expected to find based on our research questions and literature review) and emergent themes (new insights that arose directly from the data). The flexibility of nonlinear coding was instrumental in accommodating the dynamic nature of qualitative data, ensuring that we did not overlook any significant aspects.

For organizing and interpreting the data, we used the SWOT (Strengths, Weaknesses, Opportunities, Threats) analysis framework. This strategic planning tool was particularly effective in categorizing the findings into distinct but interconnected categories, facilitating a structured yet comprehensive understanding of the HIIM field's challenges and prospects.

Through this meticulous data analysis process, we were able to gain a comprehensive understanding of the challenges and dynamics in the field of HIIM, thereby aligning our findings with the study's overarching objectives.

Ethical considerations

The study was approved by the Research Ethics Committee (REC) at the University of Hail (Approval No: H-2023-405). Ethical standards were rigorously upheld throughout the research process. Participants were provided with detailed information about the study, and informed consent was obtained. Measures were taken to ensure confidentiality and privacy, with all data anonymized to protect participants' identities. The research prioritized beneficence, ensuring participant well-being and minimizing potential harm. Participation was voluntary, with provisions for withdrawal at any stage. Transparency in research processes, including methodology and data analysis, was maintained, and any potential conflicts of interest were disclosed.

## Results

Profile of key participants

The study involved interviewing 39 informants in Hail City from a wide range of professional backgrounds within the HIIM sector. These included HI, HIS technicians, computer science specialists, nursing informaticians, HIIM specialists, physicians, academicians, and both undergraduate and postgraduate students. Refer to Table 1 for detailed participant profiles.

**Table 1:** Profile of Key Participants (n = 39) HIIM: Health Informatics and Information Management that integrates health informatics (HI) and information management, focusing on managing and using information in healthcare. E-health: Electronic Health that refers to healthcare practices and services supported by electronic processes and communication, intersecting healthcare and information technology. HI: Health Informatics that is an interdisciplinary field focused on the use of biomedical data, information, and knowledge for scientific inquiry, problem-solving, and decision making to improve human health. HIS: Health Information System where systems are designed for managing healthcare data, including the handling of electronic medical records, hospital administration, and health service planning.

Study ID	Sex	Professional Background		Professional	Years of Experience	Global Training or Experience (Inside/Outside KSA)
A1	M	Undergraduate of HIIM	N/A		N/A	Saudi Arabia
A2	M	Health informatician	IT Project Manager		3	Saudi Arabia
A3	M	Health informatics; Master	E-health dept Manager		8	Saudi Arabia
A4	M	Health informatics; Master	Academician		3	Saudi Arabia
A5	M	Computer Science Specialist	Director of HI department		5	Australia
A6	M	Technician of HIS	Receptionist		7	Saudi Arabia
A7	F	Technician of HIS	Technician of Medical records		4	Saudi Arabia
A8	F	HIIM Specialist	Receptionist		9	Saudi Arabia
A9	F	Postgraduate of HI; Master	N/A		N/A	Saudi Arabia
A10	F	Postgraduate of HI; Master	N/A		N/A	Saudi Arabia
A11	M	Postgraduate of HIM; Master	N/A		2	Saudi Arabia
A12	F	Computer Science Specialist	Health Care IT Senior Manager		11	Saudi Arabia
A13	M	HIIM Specialist	Call center		7	United Kingdom
A14	F	Postgraduate of HIM; Master	Academician		3	Saudi Arabia
A15	F	Staff Nurse	Nursing informatician		8	Philippine
A16	F	Health informatics; PhD	Health informatics Consultant		13	Australia
A17	M	Staff Nurse	Administrator		11	Philippine
A18	M	Nursing Informatics; Master	Head of the nursing dept		7	Saudi Arabia
A19	M	Clinical Informatics; Master	Project Manager		9	Saudi Arabia
A20	M	Physician	Quality department Manager		13	Australia
A21	M	Undergraduate of HIIM	N/A		N/A	Saudi Arabia
A22	M	Data Analyst	Developer and analyst of data in HIM systems		7	Saudi Arabia
A23	M	HIM Senior Manager	Manager of administrative activities of HIMS		13	United States
A24	F	Data Quality Manager	Manager of data quality in HIS		11	Saudi Arabia
A25	M	Assistance Professor	Academician		6	India
A26	M	Undergraduate of HIIM	N/A		N/A	Saudi Arabia
A27	M	Health informatician	IT Project Manager		3	Saudi Arabia
A28	M	Health informatics; Master	E-health dept Manager		8	Saudi Arabia
A29	M	Health informatics; Master	Academician		3	Saudi Arabia
A30	M	Computer Science Specialist	Director of HI department		5	Australia
A31	M	Technician of HIS	Receptionist		7	Saudi Arabia
A32	F	Technician of HIS	Technician of Medical records		4	Saudi Arabia
A33	F	HIIM Specialist	Receptionist		9	Saudi Arabia
A34	F	Postgraduate of HI; Master	N/A		N/A	Saudi Arabia
A35	F	Postgraduate of HI; Master	N/A		N/A	Saudi Arabia
A36	M	Health informatics; Master	Team leader of HIS		5	Saudi Arabia
A37	M	Physician	General practitioner		7	Australia
A38	M	Computer Science Specialist	Director of IT department		10	Saudi Arabia
A39	M	Technician of HIS	Receptionist in HIM dept.		6	Saudi Arabia

The demographic composition of the participants was predominantly Saudi nationals with a diverse gender representation, although a higher proportion were males. The participants came from various professional backgrounds, reflecting a multidisciplinary nature in the field. Experience levels varied, ranging from novices to those with over a decade of experience. Several participants also had global training or experience, notably in countries such as the United States, Australia, the United Kingdom, the Philippines, and India.

Table 1 presents the profiles of key participants, including details such as study ID, sex, professional background, years of experience, and global training or experience.

SWOT analysis

The SWOT analysis, derived from the key informant interviews, highlighted various aspects of the HIIM sector in Hail City. This analysis identified strengths, weaknesses, opportunities, and threats in the field.

Strengths identified include extensive knowledge in healthcare, computer science, and information systems among professionals, the novelty of the HIIM major, professional development and motivation within the field, the presence of specialized instructors, and qualified Saudi HIIM professionals.

Weaknesses noted were the lack of specialized faculty in HIIM within universities, inadequate collaboration between HIIM professionals and other healthcare practitioners, absence of practical education at the collegiate level, and issues related to internship experiences.

Opportunities for growth and development in HIIM were seen in the potential acceleration of Electronic Health Record (EHR) implementation, high-level government support for HI initiatives, and growing job prospects with competitive salaries.

Threats included the involvement of unspecialized academicians in HI education, leading to diluted curriculum competencies and decision-making, lack of a balanced curriculum in healthcare and technical courses, HIIM specialists working in roles unrelated to their specialty, and unclearly identified career paths in HIIM.

Table 2 presents a detailed SWOT analysis, categorizing the various strengths, weaknesses, opportunities, and threats along with their respective rankings.

**Table 2:** SWOT Analysis SWOT: Strengths, weaknesses, opportunities, threats; HIIM: health informatics and information management; HI: health informatics; EHR: electronic health record

SWOT	Rank	Item
Strengths	1	Knowledge in healthcare, computer science, and information systems.
	2	New major.
	3	Professional development and motivation in HIIM.
	4	Instructors specialized in multiple fields related to HIIM.
	5	A qualified Saudi HIIM professional.
Weaknesses	1	The University lacks specialized faculty in HIIM.
	2	Inadequate collaboration between HIIM professionals and other healthcare practitioners.
	3	There's no practical education at college.
	4	Issues with the internship.
Opportunities	1	Accelerate the implementation of EHR in public and private health sectors.
	2	High-level and top leadership support from the government for HI.
	3	Growing jobs and salaries.
Threats	1	Unspecialized academicians are involved in HI education, leading curriculum competencies and decision-making.
	2	A balanced curriculum is lacking in healthcare and technical courses.
	3	HIIM specialists are working in roles unrelated to their specialty.
	4	Career paths are not clearly identified in HIIM.

## Discussion

The evolution of multidisciplinary approaches in HI technologies marks a significant transformation in the field. Information systems offer numerous opportunities and methodologies that enhance the medical informatics field, facilitating the integration of technology into healthcare practices [15]. This progress provides practitioners with a broad skill set, essential for addressing the complex challenges in healthcare and fostering adaptability and innovation [16].

Despite these advancements, challenges remain. A notable concern is the shortage of specialized HIIM faculty, which potentially affects the quality of education and the development of essential skills [17]. This issue is exacerbated by the need for enhanced collaboration between HIIM professionals and healthcare practitioners, an objective that aligns with Saudi Arabia's Vision 2030 and its focus on healthcare digitization [18].

The study corroborates findings by Alhur and others, highlighting substantial gaps in practical education and internship experiences in HIIM [19]. These deficiencies underscore the need for practical training to prepare students for the complexities of the HIIM profession [20]. Additionally, understanding user perceptions, particularly among nursing staff regarding electronic medical records, provides valuable insights into factors influencing technology acceptance and usability [21]. This knowledge is crucial for addressing challenges in implementing digital health systems.

Opportunities for HIIM professionals are burgeoning, particularly with the increasing adoption of EHRs and strong governmental support [22]. These developments align with the national digital transformation strategy and promise to enhance professional opportunities within the field.

Yet, the sector faces numerous threats. The involvement of underqualified academicians, gaps in curricula that integrate healthcare and technical courses inadequately, and the practice of specialists working outside their areas of expertise are significant barriers [23]. Moreover, the absence of well-defined career pathways complicates professional development for HIIM specialists [24]. Tackling these challenges is crucial for the progression of HIIM professionals and the field's overall advancement.

Study's limitations 

This research, aimed at exploring the field of HIIM in Hail City, Saudi Arabia, encounters several limitations that are important to consider. Firstly, the geographical scope of the study was confined to Hail City. This limitation may affect the broader applicability of the findings to different regions, where HIIM experiences and challenges can vary significantly. Additionally, the study's participant pool consisted of thirty-nine informants, which, while providing a range of viewpoints, suggests that a larger and more diverse sample might have yielded a richer understanding of the field.

Another potential limitation is the bias in self-reporting, a common issue in interview-based studies. Participants' responses could be influenced by their personal experiences or their desire to present themselves in a certain way. Furthermore, given the rapidly evolving nature of the HIIM field, the relevance of this study's findings might be limited over time. Technological advancements and changes in healthcare policies could rapidly alter the context described in this study.

The study employed NVivo for data analysis, a tool that, while effective, has limitations in qualitative analysis. The researchers' perspectives inherently influence the interpretation of data. Additionally, the study predominantly relies on qualitative data, which, despite offering depth, lacks the statistical strength of quantitative approaches. This reliance limits the ability to make broader generalizations from the findings.

Lastly, the study focused on specific areas of HIIM, potentially overlooking other influential factors, such as economic or political elements. Recognizing these limitations is key to understanding the scope of the study and the context within which its findings should be interpreted. Future research in this area could benefit from a broader geographical range, a larger and more diverse participant group, the use of quantitative methods, and an exploration of additional dimensions that impact HIIM.

Recommendations

To support the expansion and advancement of HIIM, several recommendations are proposed. It is essential to gather accurate data on the current HIIM workforce to understand workforce composition and knowledge gaps for targeted interventions. Training programs should be created to enhance physicians' abilities and acceptance of HIT within healthcare organizations, ensuring effective integration and utilization. Collaborating with healthcare standards bodies is key to validating and adapting universal competency domains for integration into postgraduate medical education. This will equip doctors with essential HIIM skills. Access to resources such as computer labs and EMR systems is necessary for students to gain hands-on experience with HIIM concepts. Assessing teaching effectiveness through student assessments, feedback, and curriculum alignment will improve teaching methodologies. Providing research opportunities for undergraduate students and encouraging their active involvement as co-authors will enhance their research skills. Assistance in creating detailed career and educational plans in the HIIM field will guide students' professional development. Promoting collaboration across departments and colleges will facilitate knowledge transfer and interdisciplinary research. Finally, fostering student engagement through mentoring programs, networking opportunities, and extracurricular activities will boost motivation and success in HIIM. The successful implementation of these recommendations depends on collaborative efforts among all stakeholders involved in HIIM.

## Conclusions

This research paper provides a comprehensive SWOT analysis of HIIM. It delves into the strengths, weaknesses, opportunities, and threats associated with HIIM, particularly in the context of Hail City, Saudi Arabia. The study recognizes the multidisciplinary nature of HIIM, encompassing expertise in healthcare, computer science, and information systems. The presence of highly qualified professionals in Hail City significantly contributes to the advancement of HIIM. The opportunities arising from the KSA 2030 Vision and the ongoing digital revolution are bolstering the field. However, the field faces challenges such as limited university faculty resources, insufficient collaboration between healthcare practitioners and HIIM professionals, and gaps in practical education and internship programs. Opportunities identified include the acceleration of electronic health record implementation, governmental support, expanding job markets, and the establishment of a national health information center. Threats to the field include the involvement of underqualified academicians, an unbalanced curriculum, HIIM specialists working in roles unrelated to their specialty, and unclear career pathways. Addressing these challenges is crucial for realizing the full potential of HIIM and ensuring its growth and sustainability.
